# Preterm birth alters the development of cortical microstructure and morphology at term-equivalent age

**DOI:** 10.1016/j.neuroimage.2021.118488

**Published:** 2021-11

**Authors:** Ralica Dimitrova, Maximilian Pietsch, Judit Ciarrusta, Sean P. Fitzgibbon, Logan Z.J. Williams, Daan Christiaens, Lucilio Cordero-Grande, Dafnis Batalle, Antonios Makropoulos, Andreas Schuh, Anthony N. Price, Jana Hutter, Rui PAG Teixeira, Emer Hughes, Andrew Chew, Shona Falconer, Olivia Carney, Alexia Egloff, J-Donald Tournier, Grainne McAlonan, Mary A. Rutherford, Serena J. Counsell, Emma C. Robinson, Joseph V. Hajnal, Daniel Rueckert, A. David Edwards, Jonathan O'Muircheartaigh

**Affiliations:** a Centre for the Developing Brain, School of Biomedical Engineering and Imaging Sciences, King's College London, London, United Kingdom; b Department of Forensic and Neurodevelopmental Sciences, Institute of Psychiatry, Psychology and Neuroscience, King's College London, London, United Kingdom; c Centre for Functional MRI of the Brain (FMRIB), Welcome Centre for Integrative Neuroimaging, Nuffield Department of Clinical Neurosciences, University of Oxford, Oxford, United Kingdom; d Department of Electrical Engineering, ESAT/PSI, KU Leuven, Belgium; e Biomedical Image Technologies, ETSI Telecomunicación, Universidad Politécnica de Madrid and CIBER-BBN, Madrid, Spain; f Biomedical Image Analysis Group, Department of Computing, Imperial College London, London, United Kingdom; g MRC Centre for Neurodevelopmental Disorders, King's College London, London, United Kingdom; h South London and Maudsley NHS Foundation Trust, London, United Kingdom; i Faculty of Informatics and Medicine, Klinikum rechts der Isar, Technical University of Munich, Munich, Germany

**Keywords:** Preterm birth, Cortical development, Neonatal neuroimaging, Heterogeneity, BSID-III, Bayley III Scales of Infant and Toddler Development, dHCP, developing Human Connectome Project, dMRI, diffusion MRI, FA, fractional anisotropy, fICVF, intracellular volume fraction, GA, gestational age, GPR, gaussian process regression, MAE, mean absolute error, MD, mean diffusivity, MRI, magnetic resonance imaging, MSE, mean squared error, MSM, multimodal surface matching, NODDI, Neurite Orientation Dispersion and Density Imaging, ODI, orientation dispersion index, PALM, Permutation Analysis of Linear Models, PMA, postmenstrual age, PWMLs, punctate WM lessions, RF, random forest, SA, surface area, TEA, term-equivalent age, WM, white matter

## Abstract

**Introduction:**

The dynamic nature and complexity of the cellular events that take place during the last trimester of pregnancy make the developing cortex particularly vulnerable to perturbations. Abrupt interruption to normal gestation can lead to significant deviations to many of these processes, resulting in atypical trajectory of cortical maturation in preterm birth survivors.

**Methods:**

We sought to first map typical cortical micro- and macrostructure development using *invivo* MRI in a large sample of healthy term-born infants scanned after birth (*n* = 259). Then we offer a comprehensive characterization of the cortical consequences of preterm birth in 76 preterm infants scanned at term-equivalent age (37–44 weeks postmenstrual age). We describe the group-average atypicality, the heterogeneity across individual preterm infants, and relate individual deviations from normative development to age at birth and neurodevelopment at 18 months.

**Results:**

In the term-born neonatal brain, we observed heterogeneous and regionally specific associations between age at scan and measures of cortical morphology and microstructure, including rapid surface expansion, greater cortical thickness, lower cortical anisotropy and higher neurite orientation dispersion. By term-equivalent age, preterm infants had on average increased cortical tissue water content and reduced neurite density index in the posterior parts of the cortex, and greater cortical thickness anteriorly compared to term-born infants. While individual preterm infants were more likely to show extreme deviations (over 3.1 standard deviations) from normative cortical maturation compared to term-born infants, these extreme deviations were highly variable and showed very little spatial overlap between individuals. Measures of regional cortical development were associated with age at birth, but not with neurodevelopment at 18 months.

**Conclusion:**

We showed that preterm birth alters cortical micro- and macrostructural maturation near the time of full-term birth. Deviations from normative development were highly variable between individual preterm infants.

## 1 Introduction

The period encompassing the last trimester of gestation and early postnatal life is characterized by dynamic progressive and regressive cellular events that shape the developing cortex, establishing the fundamental anatomical organization of the human brain and promoting the emergence of cognitive functioning. Rapid dendritic arborization, synaptogenesis, ingrowth of thalamocortical afferents combined with the disappearance of radial glia and the dissolution of the subplate drive cortical growth and a significant increase in cytoarchitectural complexity (Sidman and Rakic 1973; Kostović and Jovanov-Milošević 2006). The cortex is particularly vulnerable to perturbations during this time, with deviations and/or interruptions to one or multiple of these processes predisposing to atypical development and possible long-lasting behavioral consequences (Volpe 2019).

Preterm birth, or being born before 37 weeks gestational age (GA), represents a significant risk for adverse neurodevelopment (Marlow et al., 2005). While white matter (WM) injury has been considered as the core pathology associated with preterm birth, it is now evident that altered cortical development might underlie some of the cognitive deficits in preterm birth survivors (Volpe 2005; Miller and Ferriero 2009; Rathbone et al., 2011; Fleiss et al., 2020). Magnetic Resonance Imaging (MRI) studies show that preterm infants have altered cortical growth, expansion, folding and microstructure at term-equivalent age (Ajayi-Obe et al., 2000; Ball et al., 2013, 2020; Engelhardt et al., 2015; Makropoulos et al., 2016; Bouyssi-Kobar et al., 2018; Kline et al., 2019), with a correlation between early cortical ‘dysmaturation’ and later cognitive abilities (Kline et al., 2020). Previous work, however, has primarily focused on describing cortical development in the *preterm* brain exclusively, with little reference to normative development, or has been limited by small term-born samples. A comprehensive characterization of the cortical sequelae of preterm birth compared to normative development is necessary to better understand the role of cortical (dys)maturation in the encephalopathy of prematurity.

In this study, we aimed to describe typical cortical micro- and macrostructural maturation using *in vivo* structural and diffusion MRI in a large sample of term-born infants scanned during the neonatal period, to then test how preterm birth shapes the developing cortex. Early brain development is highly variable between individuals, and even those belonging to the same risk or clinical ‘group’ often show heterogeneous pattern of deviations from typical maturation (Dimitrova et al., 2020) and follow diverse neurocognitive trajectories (Johnson et al., 2018; Bogičević et al., 2021). Therefore, to offer a more precise understanding of the early cortical consequences of preterm birth, we not only characterize the group-average abnormality, but also capture the heterogeneity across the preterm population.

## 2 Materials & methods

### 2.1 Participants

509 infants, of which 109 preterm born, were recruited for the developing Human Connectome Project (dHCP, approved by the National Research Ethics Committee; REC: 14/Lo/1169). Infants were scanned at term-equivalent age (TEA, 37 – 45 weeks postmenstrual age (PMA)) during natural unsedated sleep at the Evelina London Children's Hospital. Infant preparation and imaging have been described previously in detail (Hughes et al., 2017). Exclusion criteria for the term-born sample included admission to neonatal intensive care unit or a significant intracranial abnormality detected on neonatal MRI scan (including acute infarction and parenchymal haemorrhage), but not punctate white matter lesions (PWMLs), small subependymal cysts/haemorrhages in the caudothalamic notch, mildly prominent ventricles or widening of the extra-axial CSF (within normative variation). There were no exclusion criteria for the preterm infants, except for major congenital malformations or diagnosed genetic disorders.

### 2.2 MRI acquisition and preprocessing

MRI data were acquired using a 3T Philips Achieva scanner equipped with a dedicated 32-channel neonatal head coil and baby transportation system (Hughes et al., 2017). T_2_-weighted scans were acquired with TR/TE of 12 s/156 ms, SENSE=2.11/2.58 (both axial/sagittal) with in-plane resolution of 0.8 × 0.8 mm (1.6 mm slice thickness, 0.8 mm overlap). Images were reconstructed using super-resolution and motion correction resulting in 0.5 mm isotropic grid sizes (Cordero-Grande et al., 2018). Diffusion MRI (dMRI) data were acquired in a total of 300 volumes, sampling b-values of 400 s/mm^2^, 1000s/mm^2^ and 2600s/mm^2^ spherically uniformly distributed in 64, 88 and 128 directions, along with 20 *b* = 0 s/mm^2^ images. Protocol parameters were multiband acceleration 4, SENSE factor 1.2, partial Fourier 0.86, acquired resolution 1.5 × 1.5 mm (3 mm slice thickness, 1.5 mm overlap), TR/TE of 3800/90 ms and 4 phase-encoding directions (Hutter et al., 2018).

Structural MRI data were preprocessed using the dHCP structural pipeline (Makropoulos et al., 2018). In brief, motion-corrected, reconstructed T_2_-weighted images were corrected for bias-field inhomogeneities, brain extracted and segmented into 9 tissue classes using the Draw-EM algorithm (Makropoulos et al., 2016). Following tissue segmentation, white, pial and midthickness surfaces were extracted, inflated and projected onto native left/right surfaces (Schuh et al., 2017) which were registered to a neonatal specific 40-week dHCP surface template (Bozek et al., 2018) using multimodal surface matching (MSM) (Robinson et al., 2014, 2018). For each infant, metrics of corrected cortical thickness (curvature regressed out), pial surface area (SA), curvature and sulcation were extracted (all but SA automatically generated from the dHCP pipeline).

Diffusion MRI data were preprocessed with denoising (Veraart et al., 2016), Gibbs ringing removal (Kellner et al., 2016), B_0_ field map estimation (Andersson et al., 2003) and reconstructed to 1.5 mm isotropic resolution using a multi-shell spherical harmonics and radial decomposition signal representation with a slice-to-volume motion and distortion correction (Christiaens et al., 2021). The tensor model was fitted to the *b* = 400 and *b* = 1000 s/mm^2^ shells. Scalar maps of fractional anisotropy (FA) and mean diffusivity (MD), in μm^2^/ms (10^−3^ mm^2^/s), were calculated using MRtrix3 (Tournier et al., 2019). Neurite Orientation Dispersion and Density Imaging (NODDI) was fitted to all shells using the NODDI MATLAB toolbox (Zhang et al., 2012). Maps of Orientation Dispersion index (ODI) and neurite density index (or intracellular volume fraction, fICVF), were derived using the default settings in the NODDI toolbox option ‘*invivopreterm’* with intrinsic diffusivity fixed to 1.7 μm^2^/ms and grid search starting points adjusted to better fit newborn data by lowering the range of values considered as the fraction of the intra-neurite space from 0- 1 to 0–0.3 (Kunz et al., 2014).

Infants’ dMRI-derived maps were affine registered to native T_2_-weighted images via the mean b1000 shell using *epi_reg*, implemented in FSL (Jenkinson et al., 2002). When projecting diffusion metrics to the cortical surface, we applied partial volume correction by estimating partial voluming on a voxel level using Toblerone (Kirk et al., 2020) (*Suppl. Materials & Suppl.* Fig. 1). Following partial volume estimation and correction, diffusion maps were projected to native surface space using ribbon constrained mapping and resampled to the 40-week dHCP neonatal surface template using adaptive barycentric interpolation (implemented in Connectome Workbench, https://www.humanconnectome.org/software/connectome-workbench).

**Fig. 1:**
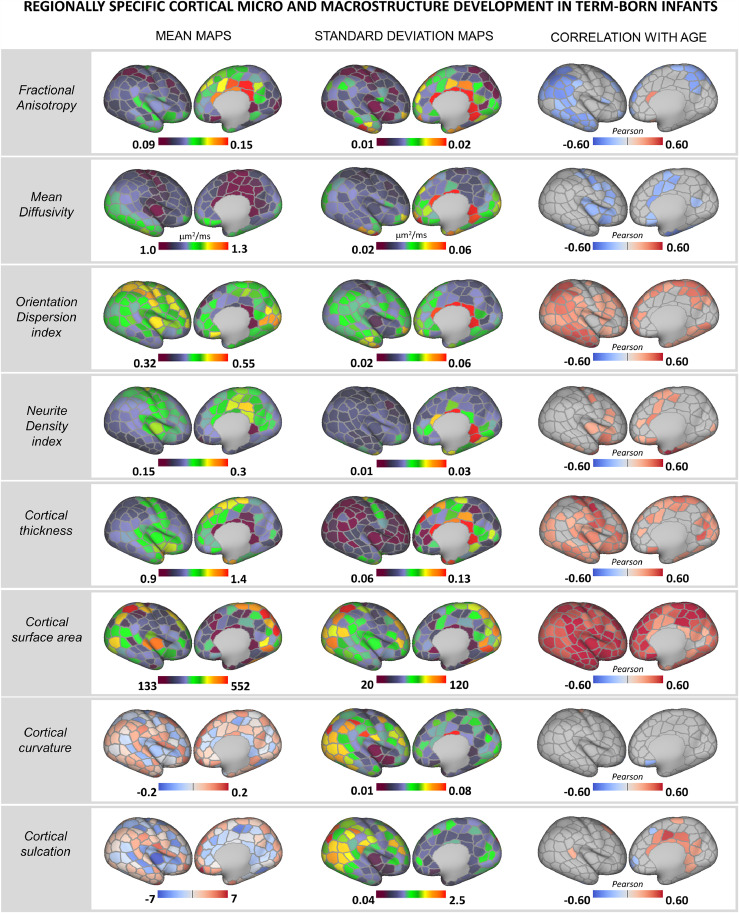
Rapid regionally specific cortical micro- and macrostructure development in term-born infants during the neonatal period. Mean and standard deviation surface maps are shown for all metrics together with Pearson's correlation coefficients for all parcels showing a significant (p_mcfwe_ < 0.05) positive (red) or negative (blue) association with age (PMA at scan). Right hemisphere depicted (Pearson's correlation coefficients for left hemisphere parcels are shown in *Suppl.*Fig. 4).

We used a two-stage quality control (in volume and surface space) to ensure no data affected by (i) motion, or with (ii) artefacts, (iii) poor dMRI to T_2_-weighted registration, (iv) unsatisfactory surface projection or registration to surface template were included (*Suppl. Materials*). The final sample comprised 259 term-born and 76 preterm infants (*Suppl.* Table 1). Cortical regions were defined using approximately equal-sized cortical parcellations with Voronoi decomposition (*n* = 143 per hemisphere). For all metrics with the exception of SA, where the sum was used, we calculated the mean value per parcel for every infant to be used for subsequent analyses.

**Table 1:** Characteristics of the study sample.

	Term infants *n = 259*	Preterm infants *n = 76*
GA at birth, *median (range)*	40.14 (37–42.14)	32.07 (23–36.86)
PMA at scan, *median (range)*	40.86 (37.43–44.71)	40.93 (37–45.14)
Postnatal weeks, *median (range)*	0.43 (0–4.86)	8.71 (0.28––19.57)
Female, *n (%)*	118 (46%)	34 (45%)
Birth weight, *median (range)*	3.36 (2.1–4.6)	1.73 (0.45–4.1)
Birth HC, *median (range)*	34.5 (30–38)	30 (20.5–36)
APGAR at 1 min, *median (range)*	9 (1–10)	7 (1–10)
APGAR at 5 min, *median (range)*	10 (6–10)	9 (1–10)
PWMLs, *n (%)*	36 (14%)	27 (36%)
Cerebellar haemorrhage, *n (%)*	–	5 (7%)
Behavioural follow-up, *No. infants (%)*	212 (82%)	58 (76%)
Age (corrected months)*, median (IQR)*	18.3 (18–18.9)	18.4 (18.2––19.2)
BSID-III Motor, *median (IQR)*	103 (97–110)	100 (94–107)
BSID-III Language, *median (IQR)*	97 (89–106)	100 (91–109)
BSID-III Cognition, *median (IQR)*	100 (95–109)	100 (95–106)

GA – Gestational age at birth; IQR – interquartile range; PMA – Postmenstrual age at scan; HC – head circumference; APGAR - Appearance, Pulse, Grimace, Activity, and Respiration score; PWMLs – punctate white matter lesions; BSID-III – Bayley Scales of Infant Development III.

### 2.3 Typical cortical development in term-born infants

#### 2.3.1 Association between age at scan and cortical microstructure and morphology

First, we described age-associated changes in cortical microstructure and morphology in the full 259 term-born sample. This was performed with Permutation Analysis of Linear Models (PALM) implemented in FSL using 10,000 permutations and a family-wise error (fwe) correction across modalities (8 metrics) and contrasts (positive/negative association with PMA at scan), including sex in the model (Winkler et al., 2014; Alberton et al., 2020). Pearson's coefficients were reported for parcels showing a significant correlation at *p*_mcfwe_ < 0.05 (*m*odality *c*ontrast *fwe* corrected).

#### 2.3.2 Predicting age at scan using cortical features

In addition, to predict PMA at scan, we used Random Forest (RF) regression in *Scikit Learn* (Pedregosa et al., 2011). The 259 term-born infants were split into training (75%) and hold-out (25%) samples (*Suppl.* Fig. 2). Model hyperparameters were tuned on the training dataset using Bayesian Optimisation search (*Scikit Learn Optimize*) under 5-fold cross validation and mean absolute error (MAE) as loss function. Optimal hyperparameters were max_depth = 35, max_features = ‘auto’, min_sample_leafs = 2, n_estimators = 1000. The RF predictions were also error-in-variables bias corrected using linear regression (Smith et al., 2019). Model performance was evaluated by estimating the MAE, mean squared error (MSE) and Spearman correlation (ρ) between true and predicted PMA at scan in the hold-out sample using the RF and error-in-variables correction models estimated on the training dataset. We examined the top 10% of the features using the default *Scikit Learn feature_importances*_.

**Fig. 2:**
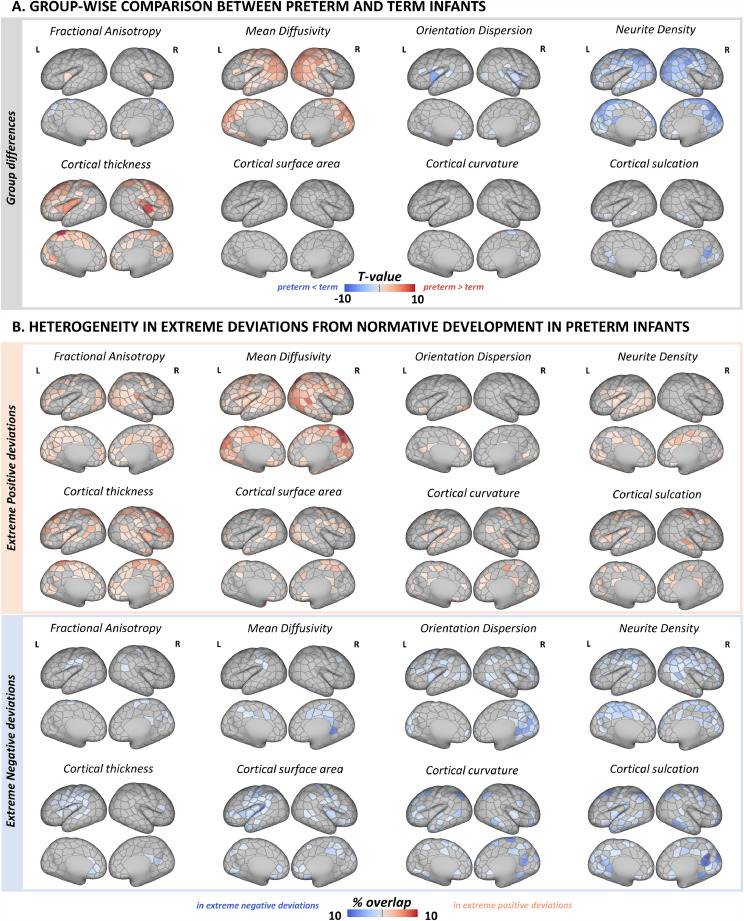
Effect of preterm birth on the developing cortex. (A) Group-level differences in cortical micro- and macrostructure between term-born and preterm infants. Depicted are only parcels that show a significant group-wise difference at p_mcfwe_ < 0.05. (B) Spatial overlap in extreme positive (red) and negative (blue) deviations from normative development in preterm infants. The overlap maps show the proportion of infants with extreme deviations (*Z* > |3.1|) from normative development for every parcel.

### 2.4 Cortical development in the preterm brain at term-equivalent age

#### 2.4.1 Group-level differences between preterm and term-born infants

Prior to testing for group mean differences (‘main effect’ of group) between term-born and preterm infants at TEA, we examined if any of the cortical features showed a significant group * PMA at scan interaction. Analyses were performed in PALM for 259 term-born and 76 preterm infants, including sex as a covariate and correcting across contrasts (slope preterm < slope term; slope preterm > slope term) and modalities (8 metrics). As a follow-up we also quantified the correlation between PMA at scan and all cortical features in the preterm sample alone (as described in Section 2.3.1).

If no significant interactions were observed, we then tested for group mean differences between term-born (*n* =259 ) and preterm infants (*n* = 76). Analyses were performed in PALM, including PMA at scan and sex as covariates in the model and correcting across contrasts (preterm < term; preterm > term) and modalities (8 metrics). We report the t-statistic for parcels indicating a significant group mean difference at *p*_mcfwe_ < 0.05.

#### 2.4.2 Mapping cortical variability in the preterm brain with Gaussian process regression

To capture the heterogeneity of cerebral development in the preterm brain, we used multioutput Gaussian process regression (GPR), a Bayesian non-parametric estimation technique, implemented in GPy (https://sheffieldml.github.io/GPy/). GPR simultaneously provides point estimates and measures of predictive confidence for every observation representing the distance of each individual from the normative mean at that point on the ‘curve’ accounting for modeled covariates. Analogous to the widely employed paediatric height and weight growth charts, this technique allows the local imaging features of individual infants to be referred to typical variation while simultaneously accounting for variables such as age and sex (Marquand et al., 2016; Marquand et al 2019; Wolfers et al., 2018; O'Muircheartaigh et al., 2020).

We first trained a GPR model to describe normative cortical development in the training term-born sample (75% of the sample: 196 infants; *Suppl.* Fig. 2) using PMA at scan and sex as predictors and FA, MD, ODI, fICVF, cortical thickness, SA, curvature and sulcation as outputs. The relationship between cortical outputs and model predictors was estimated with a sum of radial basis function, linear and white noise covariance kernels. Model hyperparameters were optimized using log marginal likelihood. Prediction performance was evaluated using the MAE between predicted and observed values derived from the hold-out term-born sample (25% of the term sample: 63 term infants). To assess the effects of preterm birth, we applied the model to 76 preterm infants scanned at TEA. A Z-score was derived for every infant in the preterm and hold-out term-born samples by estimating the difference between the model prediction and the observed value normalized by the model uncertainty (the square root of the predicted variance). To quantify extreme deviations, prior to analyses, we chose a threshold of |Z|>3.1 (corresponding to *p* < 0.001) (Dimitrova et al., 2020).

#### 2.4.3 Spatial overlap maps

The spatial prevalence of extreme negative/positive deviations across the preterm sample was examined by calculating a parcel-based percentage map of extreme deviations (number of infants with |*Z*| > 3.1 divided by the total number of infants), individually for every cortical feature.

#### 2.4.4 Atypicality indices

For every cortical feature and infant, we classified each parcel as either normative (*Z* < |3.1|) or atypical (*Z* > |3.1|) to then calculated a positive and a negative whole-cortex atypicality index. These indices capture the extreme positive/negative deviations for every infant and represent the ‘overall burden’ as a percentage of cortical parcels with extreme deviations (*Z* > |3.1|) relative to the total number of parcels. Thus, if an infant had 29 parcels with extreme negative deviations in cortical FA (*Z* < −3.1), the FA- atypicality index for this infant will be 10% (29 out of 286).

#### 2.4.5 Deviations from normative development, age at birth and behavior at 18 months

To test if age at birth influences cortical development, we examined the association between GA at birth and GPR derived Z-scores; and between GA at birth and the whole-cortex atypicality indices (in preterm infants; in hold-out term-born infants and in the combined preterm and hold-out term-born infants). To assess if deviations from normative cortical development relate to later behavior, we looked at the correlation between Bayley III Scales of Infant and Toddler Development (BSID-III) (Bayley 2006) composite motor, cognitive and language scores, collected at 18 months corrected age, and Z-scores and atypicality indices. PALM was used (as described above) for parcel-wise Z-score data; Kendall τ (for correlation) and Mann-Whitney *U* (for group differences) for the atypicality indices. Analyses were corrected for multiple comparison correction (FDR).

### 2.5 Data availability

The MRI data for the dHCP are freely available at http://www.developingconnectome.org/. dHCP surface template is available at https://brain-development.org/brain-atlases/atlases-from-the-dhcp-project/cortical-surface-atlas-bozek/. Code for MSM surface registration can be found at https://github.com/ecr05/MSM_HOCR, partial volume correction code at https://git.fmrib.ox.ac.uk/seanf/dhcp-neonatal-fmri-pipeline/-/blob/90f2f7317fff0cd809d0b1b4a400d970c1ea115c/doc/surface.md. Python code and metadata to perform the main analyses in this work are available from https://github.com/ralidimitrova/neo_cortexDev .

## 3 Results

Term and preterm infants did not differ in PMA at scan (*p* = 0.90), nor in sex distribution (*p* = 0.89) (Table 1). Distribution of GA at birth and PMA at scan are shown in *Suppl.* Fig. 3. While PWMLs were more frequently seen in the preterm sample (*p*<0.05), no infants had severe WM injury such as periventricular leuomalacia and only one preterm infant had more than 20 PWMLs. One preterm infant had cystic lesions in both thalami extending to internal capsule and left putamen, one had cerebellar hemispheric parenchymal loss accompanied by brainstem atrophy, and one had middle cerebral artery infarct (the last resulting in gross cortical injury, excluded from all group-level analyses). No group differences were observed at the 18 month follow-up (cognitive *p* = 0.99; motor *p* = 0.19; language *p* = 0.36). All, but two (one in language, one in cognitive outcome), preterm infants scored within 2 standard deviations from the population mean.

**Fig. 3:**
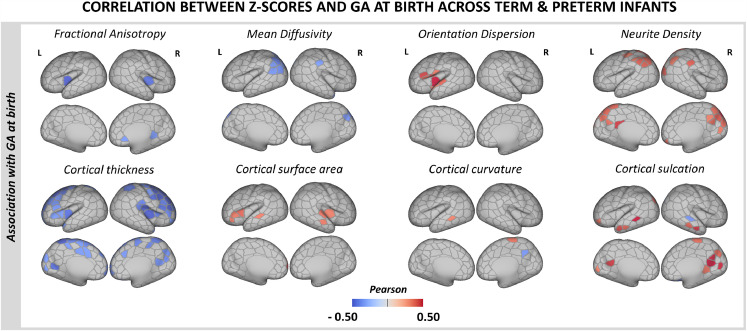
Association between deviations from normative cortical development (Z-scores) and GA at birth. Pearson's correlation coefficients are shown only for parcels showing a significant positive (red) or negative (blue) correlation with GA at birth at p_mcfwe_ < 0.05 in the combined term-born hold-out and preterm samples.

### 3.1 Rapid regionally specific micro- and macrostructural development in the term-born neonatal cortex

We observed heterogeneous and regionally specific associations between PMA at scan and MRI-derived features of cortical micro- and macrostructure during the neonatal period (Fig. 1). With increasing age at scan, lower FA was seen in the parietal, occipital and temporal lobes and lower MD in the central sulci, the anterior cingulate, the insula and in some parcels located in the frontal cortex. ODI was positively correlated with PMA across the brain, steepest in the parietal and temporal lobes, but not in the somatosensory cortex. We also observed a positive association between PMA and fICVF in the insula but also in some regions of the frontal lobe. There was a strong positive association between PMA and cortical thickness in the somatosensory, occipital, temporal, insular and portions of the parietal and frontal regions, and between PMA and SA across the whole cortex, the latter showing the strongest association with age at scan compared to all other cortical features. While curvature remained stable over the studied period, cortical sulcation was positively associated with age in the cingulate cortex. Correlations between PMA at scan and cortical features were consistent across the left and right hemisphere (Fig. 1& *Suppl.* Fig. 4).

**Fig. 4:**
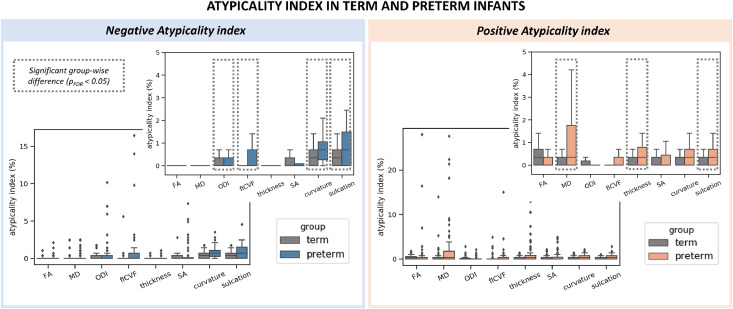
Distribution of the atypicality indices in term-born and preterm infants. The preterm brain showed higher burden of both negative (left) and positive (right) extreme deviations. Boxplots are shown with and without outliers, the latter to highlight the distribution of the indices in the two groups. Indices where a group-level difference was found are highlighted.

RF regression predicted PMA at scan in the hold-out term-born sample with MAE = 0.60, MSE = 0.56 (weeks) with a correlation between observed and predicted age of ρ = 0.85, R^2^ = 0.74 (*Suppl. Fig. 5*). Overall, SA was the ‘best’ predictor of PMA at scan, with 47% (108/229 parcels) of the 10% most important features. ODI followed with 37 parcels (16%), cortical thickness with 29 (13%), fICVF with 21 (9%), FA with 14 (6%), MD with 11 (5%), sulcation with 6 (3%) and curvature with 3 parcels (1%) (*Suppl. Fig. 6)*.

### 3.2 Cortical consequences of preterm birth at term-equivalent age

#### 3.2.1 Group-level differences between term-born and preterm infants

We did not observe a significant group * PMA at scan interaction for any of the cortical features (with the exception of a single parcel in the left hemisphere in SA, indicating steeper positive correlation with PMA in term-born compared to preterm infants). Indeed, preterm infants showed correlations between PMA at scan and cortical micro- and macrostructure (*Suppl. Fig. 7*) consistent with the associations observed in the term-born sample (Fig. 1).

At a group level, preterm infants showed higher MD and lower fICVF across a large proportion of the posterior cortex, including parcels in the parietal, occipital and temporal lobe (Fig. 2A). In the insula, preterm infants had higher FA and lower ODI compared to term-born peers. We also observed greater cortical thickness in frontal, insular and anterior parietal cortices in preterm compared to term-born infants. Apart from a few parcels, there were no group differences in SA, curvature, or sulcation.

#### 3.2.2 Heterogeneity in individual extreme deviations from normative development in the preterm sample

We observed widespread extreme positive deviations in MD and thickness, and extreme negative deviations in fICVF in preterm infants (Fig. 2B). While there was a visible overlap in parcels showing group differences in these metrics and regions with individual extreme deviations, in some cortical areas we detected only group-average alterations but no individual extreme deviations in preterm infants. And vice versa, in other parcels we observed extreme deviations on an individual infant level but not in the group-level analyses. For example, very few parcels showed group differences in FA, ODI and SA, but the spatial overlap maps were indicative of widespread individual deviations in extreme positive FA, negative ODI and positive/negative SA deviations. Overall, there were very few parcels across all metrics with more than 8% of the infants showing extreme deviations highlighting the high variability in cortical development associated with preterm birth. Term-born infants showed fewer and less overlap in extreme deviations compared to preterm infants (*Suppl. Fig. 8*). Model MAE maps are shown in *Suppl. Fig. 9.*

#### 3.2.3 Parcel-level deviations from normative development and age at birth

Lower GA at birth related to higher MD and lower fICVF in the parietal cortex (more prominent in the left hemisphere; Fig. 3.), and higher FA (bilaterally) and lower ODI (left hemisphere) in the insula. We observed a negative correlation between GA at birth and cortical thickness in the bilateral frontal and insular cortex. Very few parcels showed an association with GA at birth in the preterm or term-born samples alone (*Suppl. Fig. 10*). We observed no association between Z-scores and BSID-III neurodevelopmental scores at 18 months.

#### 3.2.4 Whole cortex atypicality indices

As a group, preterm infants had higher atypicality indices for most metrics except FA and SA (Fig. 4). On average, the preterm brain had a higher proportion of extreme negative deviations in ODI, fICVF, curvature and sulcation, as well as a higher proportion of extreme positive deviations in MD, thickness and sulcation (all at p_FDR_ < 0.05). Term-born infants did not have higher atypicality index in any of the cortical features we studied.

Lower GA at birth was related to a higher proportion of extreme negative deviations in ODI (τ = −0.15), fICVF (τ = −0.26), curvature (τ = −0.24) and sulcation (τ = −0.23; all at p_FDR_ < 0.05). Being born earlier was also associated with a higher proportion of extreme positive deviations in MD (τ = −0.24), thickness (τ = −0.20) and sulcation (τ = −0.19; all at p_FDR_ < 0.05).

After multiple comparison correction, we observed no significant associations between the atypicality indices and BSID-III scores at 18 months in the preterm sample alone, nor in the combined term-born hold-out and preterm samples.

## 4 Discussion

During early brain development, the cortex undergoes rapid changes that establish the fundamental anatomical organization of the brain. Within the period between 37 and 45 weeks PMA, we observed regionally specific micro- and macrostructural cortical maturation in a large cross-sectional sample of term-born infants. Abrupt exposure to extrauterine environment through preterm birth resulted in atypical cortical microstructure and growth at TEA, with marked variability in individual deviations from normal development. Deviations in regional cortical maturation were associated with GA at birth but not with neurodevelopment at 18 months.

### 4.1 Dynamic and spatially heterogeneous maturation of the term-born neonatal cortex

By the end of full-term gestation, neuronal migration is mostly completed, thalamocortical afferents have reached their cortical targets and the basic columnar organization is largely established (Sidman and Rakic 1973; Kostović et al., 2002; Kostović and Jovanov-Milošević 2006; Paredes et al., 2016). After birth, the dramatic increase in dendritic arborization and synapse production underlies the development of a more complex cytoarchitecture and contributes to rapid cortical growth (Marin-Padilla 1967; Huttenlocher and Dabholkar 1997; Volpe 2019). During this period, we observed increasingly more complex microstructural ‘geometry’ in the neonatal term-born brain. This was captured by lower anisotropy (FA↓) and higher neurite orientation dispersion index (ODI↑) with increasing age at scan in the parietal, occipital and temporal regions, with increasing ODI also evident in the frontal lobe. Higher neurite density index (fICVF↑) and lower tissue water content (MD↓) were observed in somatosensory and insular cortices. These associations with age suggest regionally and temporally asynchronous cortical maturation after birth (Yu et al., 2016; Batalle et al., 2019; Ouyang et al., 2019; Fenchel et al., 2020). The newborn brain is exposed to an environment rich in sensory stimuli that promotes synaptogenesis and dendritic arborisation postnatally, especially in sensory areas (Huttenlocher 1990; Huttenlocher and Dabholkar 1997), which might account for the strong age associations we observed in these regions. We described different spatiotemporal patterns of SA and cortical thickness growth. While surface expansion was very rapid and relatively uniform across the developing cortex, cortical thickness showed a strong positive association with PMA at scan in the somatosensory, temporal, occipital, insular and parts of the frontal lobe. We observed a stronger correlation between PMA and SA compared to PMA and cortical thickness (Lyall et al., 2015; Jha et al., 2019) and proportionally, SA features were the ‘best’ predictors of age at scan in our RF model. Very little association with curvature and sulcation was observed, suggesting that folding patterns are largely established by full-term gestation (Chi et al., 1977; Hill et al., 2010; Meng et al., 2014; Li et al., 2015; Batalle et al., 2019; Fenchel et al., 2020). These findings further support the complex asynchronous pattern of cortical growth in both primary and association regions during early postnatal development (Jha et al., 2019).

### 4.2 Preterm birth alters the developing cortex at term-equivalent age

The dynamic nature and complexity of the cellular events that take place in the last trimester make the developing cortex particularly vulnerable to perturbations (Volpe 2019). A number of studies have characterized the developmental trajectory of cortical microstructure in the *preterm* brain, but only a few offer a direct reference against a large normative dataset (McKinstry et al., 2002; Ball et al., 2013, 2020; Eaton-Rosen et al., 2015, 2017; Smyser et al., 2016; Yu et al., 2016; Batalle et al., 2019; Ouyang et al., 2019). Our results indicate that the *preterm* cortex exhibits more ‘immature’ microstructure at the time of full-term birth. Specifically, we show that *on average* preterm infants have increased tissue water content (MD↑) and reduced neurite density index (fICVF↓) in the occipital, parietal, temporal and the somatosensory cortex compared to term-born peers. Group differences in cortical microstructural ‘geometry’ were evident in a few parcels, mainly in the insular region, suggesting reduced neurite orientation complexity (FA↑ and ODI↓). GA at birth was negatively associated with MD and positively with fICVF in the parietal cortex, while in the insular cortex increased GA related to lower FA and higher ODI. The exact cellular mechanisms underlying this altered microstructure development are unknown. Dysmaturation of the neurons in the subplate, that offer initial targets and guidance of thalamocortical afferents before they reach the cortical plate may have a contributing role (Volpe 1996; Volpe 2019). Disrupted dendrite and spine formation, or a general reduced morphological complexity of cortical neurons, is another possible cause (Dean et al., 2013).

Unlike cortical microstructure, which appeared more ‘immature’, on average, preterm infants had thicker cortices in the somatosensory, frontal and insular regions. We observed a negative association between cortical thickness and GA at birth in most of these regions, an association that seems to persist into infancy (Jha et al., 2019). Greater cortical thickness in preterm individuals has been observed in childhood and adolescence, arguing atypical trajectory of cortical thinning as differences with term-born controls substantially decrease in early adulthood (Mürner-Lavanchy et al., 2014; Nam et al., 2015). Although the exact mechanisms, either compensatory or pathological, behind this atypical cortical growth in the preterm brain are largely unknown, they likely include altered neuronal differentiation or modification/pruning of early overproduced neurons and their processes in the cortical plate. We did not observe group-level differences in SA as reported by others (Lax et al., 2013; Engelhardt et al., 2015; Zhang et al., 2015), nor did we see a strong correlation between SA and GA at birth (Jha et al., 2019), apart from the insular cortex. Compared to previous work focusing on extremely/very preterm infants (Ajayi-Obe et al., 2000), our sample comprised ‘healthier’ preterm infants, a large proportion of which were moderate-to-late preterm (32 < GA < 37 w, *Suppl.* Fig. 3). This could partly explain why we did not observe atypical surface expansion. SA and cortical thickness are genetically independent, determined by distinct cellular processes (Rakic 1995; Panizzon et al., 2009) and follow different and regionally heterogeneous developmental trajectories (Lyall et al., 2015). While the number of cortical minicolumns established during embryonic development is believed to determine SA, the number, size and density of neurons and their processes within each minicolumn produced during the foetal and perinatal period, are thought to impact cortical thickness (Rakic 1995, 2009). The timing of the abrupt exposure to extrauterine environment might explain why cortical thickness appears more affected than SA but also why we did not observe group differences in SA between term-born and preterm infants. Given that SA is a better determinant of brain/cortical volume and size compared to cortical thickness (Pakkenberg and Gundersen 1997; Im et al., 2008), the lack of atypical surface expansion here is consistent with a previous study of this cohort, showing that the development of total tissue /cortical volume in this preterm sample was largely normative (Dimitrova et al., 2021).

Interestingly, while group mean differences were present in several measures of cortical morphology and microstructure, there was no cross-sectional evidence of different trajectories of cortical development between term-born and preterm infants. This suggests that the atypical development we observed in preterm infants at TEA might originate from the initial abrupt exposure to extrauterine environment during the preterm period, but also shows no evidence of further ‘decline’ or any compensatory mechanism taking place at TEA. Further longitudinal studies are crucial to determine this and capture the variable developmental trajectories that individual preterm infants follow.

### 4.3 Group-average atypicality and individual variability in the preterm cortex at term-equivalent age

While we show a good agreement between group-wise analyses and spatial overlap maps in extreme deviations from the normative development in MD, fICVF and thickness, high variability across individual preterm infants was evident in all metrics (Fig. 2). Very few cortical parcels had more than 8% of the preterm group with extreme deviations in a given metric. These findings are consistent with our previous work describing heterogeneous volumetric and whole-brain microstructure development at TEA following preterm birth (Dimitrova et al., 2020, 2021). This highlights that group level understanding of the preterm brain disguises a large degree of individual variability, that may be important to single infants. Preterm infants were more likely to show a higher proportion of parcels with extreme deviations from normative development (atypicality indices) compared to term-born infants (Fig. 4). Being born earlier was associated with a higher proportion of parcels with extremely low (*Z* < −3.1) ODI, fICVF, curvature and sulcation, and with more parcels with extreme high (*Z* > 3.1) MD, thickness and sulcation. This argues that when reaching the time of full-term birth, the cortex is more atypical in those born at lower GA. While we did not detect mean differences in curvature and sulcation between the two groups, preterm infants were more likely to show higher loading of extreme deviations from the normative curvature and sulcation compared to term-born infants. Atypical folding pattern has been previously reported in preterm infants (Engelhardt et al., 2015; Zhang et al., 2015) and our study highlights the high variability associated with it.

### 4.4 Cortical deviations were not associated with later neurodevelopment

Preterm birth, especially before 25 weeks GA, has been linked to poor outcome in childhood (Wood et al., 2000; Marlow et al., 2005), with a strong correlation between degree of prematurity and later cognitive functioning (Bhutta et al., 2014). Several cortical features including curvature, SA and cortical microstructure have shown promise as biomarkers predictive of later neurodevelopment in preterm infants (Kapellou et al., 2006; Rathbone et al., 2011; Ball et al., 2013; Kline et al., 2020). While we report atypical cortical development in the preterm brain at TEA, we did not observe an association between individual deviations from normative development and 18-months follow-up, or a strong correlation between cortical features and GA at birth in preterm infants. Compared to previous datasets primarily comprising extremely and/or very preterm infants, including infants with poor outcome (Ball et al., 2013; Kline et al., 2020, and Parikh 2020), our sample also included moderate-to-late preterm infants and all infants, but two, showed normative outcome at 18 months (Table 1). The small number of infants born extremely (GA < 28, *n* = 13) or very (28 < GA < 32, *n* = 24; *Suppl.* Fig. 3) preterm did not allow to explore this relationship in further subgroups based on degree of prematurity. However, in a previous study of this cohort, we found an association between overall atypicality index of whole-brain microstructure, mainly in the developing WM, and outcome (Dimitrova et al., 2020). In the preterm brain at TEA, even in the lack of overt injury, WM dysmaturation may have a stronger impact on later behavior compared to deviations in cortical development (Woodward et al., 2006).

### 4.5 Limitations

Direct correlations between imaging and histological examinations of human brain development are sparse (Trivedi et al., 2009; Dean et al., 2013; Huang et al., 2013), and caution is crucial when contributing changes in any diffusion metric to a specific biological process (Alexander et al., 2007; Kroenke 2018; Kamiya et al., 2020). Here we combined DTI and NODDI, the two most widely applied dMRI models in the study of *cortical* microstructure. DTI is highly sensitive to underlying microstructure, however, inherently lacks specificity. In particular, FA is modulated by multiple factors including dendritic/axonal density, number, orientation dispersion and partial volume contamination, creating a considerable challenge attributing changes in FA to (a) specific aspect(s) of the underlying architecture (Jones et al., 2013). While NODDI promises higher biological specificity, the model carries several limitations including the absence of any direct intrinsic diffusivity estimation. The decision to fix the intra and extracellular parallel diffusivity to the same predetermined value might represent a major source of systematic oversimplification and lead to a potential bias in parameter estimation reducing their promised specificity (Jelescu et al., 2016; Jelescu and Budde 2017). While recent work has shown significant promise (Guerrero et al., 2019), the optimal values for model parameters used in the study of the *developing* cortex are yet to be established. We chose to use the default ‘*invivopreterm’* setting in NODDI to be consistent with previous studies (Batalle et al., 2019; Ball et al., 2020; Fenchel et al., 2020). However, more work is essential to validate the estimation of the model parameters and their biological accuracy.

During the neonatal period, brain morphology is rapidly changing, the cortex is only approximately 1.1 mm thick and tissue contrast changes dramatically with maturation (Makropoulos et al., 2018; O'Muircheartaigh et al., 2020). These make image registration and segmentation of the developing brain particularly challenging. Compared to the traditional volume-based registration, the surface-based method we used is driven by geometric features of cortical shape and thus offers superior inter-subject cortical alignment (Robinson et al., 2014, 2018; Bozek et al., 2018) and lessen CSF contamination (Glasser et al., 2016). To further ensure partial volume effects are minimized, we applied surface-based partial volume correction to the dMRI data. Head motion is another challenge in pediatric imaging, that could result in blurring of the GM/WM boundary. Even subtle head motion may systematically bias the estimation of MRI-derived parameters of cortical structure including mean curvature and cortical thickness (Reuter et al., 2015; Alexander-Bloch et al., 2016; Savalia et al., 2017). While we cannot completely exclude motion as a confound in the current analyses, we used a conservative two-stage QC to ensure only data of good quality and successful preprocessing were included, and thus increase our confidence that we report biological effects. It is important that future analyses incorporate an estimate of in-scanner head motion or consider additional retrospective motion correction techniques (Vecchiato et al., 2021). Lastly, to characterize cortical development during the neonatal period, we described the association between increasing age and metrics of cortical morphology and microstructure. However, the cross-sectional nature of the data limits our ability to map the ‘true’ developmental trajectory of these metrics and future longitudinal analyses are essential to establish this.

## 5 Conclusion

We described rapid and spatially varying cortical micro- and macrostructure maturation in the term-born neonatal brain. We also showed that abrupt interruption to gestation through preterm birth alters cortical development near the time of full-term birth and that deviations from normative development are variable across individual preterm infants.

## Data and code availability statement

Data and code availability are discussed in Section 2.5. of the paper. The MRI data for the dHCP are freely available at http://www.developingconnectome.org/. dHCP surface template is available at https://brain-development.org/brain-atlases/atlases-from-the-dhcp-project/cortical-surface-atlas-bozek/. Code for MSM surface registration can be found at https://github.com/ecr05/MSM_HOCR, partial volume correction code at https://git.fmrib.ox.ac.uk/seanf/dhcp-neonatal-fmri-pipeline/-/blob/90f2f7317fff0cd809d0b1b4a400d970c1ea115c/doc/surface.md. Python code and metadata to perform the main analyses in this work are available from https://github.com/ralidimitrova/neo_cortexDev .

## Funding

The dHCP project was funded by the European Research Council (ERC) under the European Union 10.13039/501100004963Seventh Framework Programme [FR/2007–2013] /ERC Grant Agreement no. 319,456. The results leading to this publication have received funding from the Innovative Medicines Initiative 2 Joint Undertaking under grant agreement No 777,394 for the project AIMS-2-TRIALS. This Joint Undertaking receives support from the European Union's Horizon 2020 research and innovation program and EFPIA and AUTISM SPEAKS, Autistica, SFARI. The authors acknowledge infrastructure support from the National Institute for Health Research (NIHR) Biomedical Research Centre at Guy's and St Thomas’ Hospitals NHS Foundation Trust. The study was supported in part by the Wellcome Engineering and Physical Sciences Research Council Centre for Medical Engineering at King's College London [grant WT 203,148/Z/16/Z] and the Medical Research Council (UK) [grant MR/K006355/1]. D.C. is supported by the Flemish Research Foundation (FWO; grant number 12ZV420N). J.O. is supported by a Sir Henry Dale Fellowship jointly funded by the Wellcome Trust and the Royal Society [grant 206,675/Z/17/Z]. G.M. received support from the Sackler Institute for Translational Neurodevelopment at King's College London and from National Institute for Health Research (NIHR) Maudsley Biomedical Research Centre (BRC). J.O., A.D.E. and G.M. received support from the Medical Research Council Centre for Neurodevelopmental Disorders, King's College London [grant MR/N026063/1]. The views expressed are those of the authors and not necessarily those of the NHS, the NIHR, the Department of Health and IMI 2JU.

## CRediT authorship contribution statement

**Ralica Dimitrova:** Conceptualization, Data curation, Formal analysis, Investigation, Methodology, Software, Validation, Visualization, Writing – original draft, Writing – review & editing. **Maximilian Pietsch:** Conceptualization, Formal analysis, Methodology, Software, Validation, Visualization. **Judit Ciarrusta:** Conceptualization, Methodology, Investigation, Writing – original draft. **Sean P. Fitzgibbon:** Investigation, Methodology, Resources, Software, Validation. **Logan Z.J. Williams:** Investigation, Methodology, Resources, Validation. **Daan Christiaens:** Data curation, Investigation, Resources, Software. **Lucilio Cordero-Grande:** Data curation, Investigation, Resources, Software. **Dafnis Batalle:** Methodology, Resources, Software. **Antonios Makropoulos:** Methodology, Resources, Software. **Andreas Schuh:** Methodology, Resources, Software. **Anthony N. Price:** Data curation, Methodology, Investigation, Resources. **Jana Hutter:** Methodology, Investigation, Resources. **Rui PAG Teixeira:** Methodology, Investigation, Resources. **Emer Hughes:** Data curation, Methodology, Investigation, Resources. **Andrew Chew:** Data curation, Investigation, Resources. **Shona Falconer:** Data curation, Investigation, Resources. **Olivia Carney:** Data curation, Investigation, Resources. **Alexia Egloff:** Data curation, Investigation, Resources. **J-Donald Tournier:** Investigation, Methodology, Resources. **Grainne McAlonan:** Funding acquisition, Investigation, Supervision, Writing – review & editing. **Mary A. Rutherford:** Data curation, Resources, Supervision, Writing – review & editing. **Serena J. Counsell:** Data curation, Resources, Supervision, Writing – review & editing. **Emma C. Robinson:** Methodology, Software, Supervision, Validation. **Joseph V. Hajnal:** Funding acquisition, Methodology, Resources. **Daniel Rueckert:** Funding acquisition, Methodology, Resources. **A. David Edwards:** Conceptualization, Funding acquisition, Methodology, Resources, Supervision, Validation, Writing – original draft, Writing – review & editing. **Jonathan O'Muircheartaigh:** Conceptualization, Methodology, Resources, Software, Supervision, Validation, Writing – original draft, Writing – review & editing.

## Appendix Supplementary materials

Image, application 1
